# Editorial: Understanding oral health challenges in pediatric and adult cancer care

**DOI:** 10.3389/fonc.2026.1884860

**Published:** 2026-06-16

**Authors:** Rogelio Gonzalez-Gonzalez, Kumar Chandan Srivastava

**Affiliations:** 1 Department of Oral and Maxillofacial Surgery & Diagnostic Sciences, College of Dentistry, Jouf University, Sakaka, Saudi Arabia; 2 Department of Oral Medicine & Radiology, Saveetha Institute of Medical and Technical Sciences (SIMATS), Saveetha Dental College and Hospital, Saveetha University, Chennai, Tamil Nadu, India

**Keywords:** digital health, oral microbiome, oral mucositis, pediatric oncology, supportive care

## Introduction

The interaction between the oral microbiome and the systemic response to oncological treatment is gaining increasing relevance in personalized medicine (1). Regulation of the tumor microenvironment by host immunity is well documented; in this scenario, the involvement of the microbiota in pharmacological efficacy—a field known as pharmacomicrobiomics—is redefining precision oncology (2). Current evidence shows that bacterial communities not only coexist with the tumor, but that their metabolic pathways directly modulate the pharmacokinetics and pharmacodynamics of antineoplastic agents (2).

In this regard, Fu et al. provided key findings in the management of triple-negative breast cancer (TNBC), demonstrating that specific oral microbial signatures—characterized by enrichment of Lactobacillus and Neisseria—allow for the prediction of favorable responses to neoadjuvant chemotherapy (3). These results reflect a complex biological interaction, in which the microbiome modulates tumor progression and therapeutic response through two main mechanisms: systemic immunoinflammatory regulation and remodeling of the tumor microenvironment (1). In contrast, oral dysbiosis—composed predominantly of Prevotella, Clostridia, and Bacteroidetes genera—induces secretion of proinflammatory mediators such as IL-8 and CXCL1 (1). This signaling alters the immune niche by suppressing infiltration and effector activity of CD8+ T lymphocytes, thereby compromising the efficacy of regimens based on epirubicin, cyclophosphamide, and paclitaxel (1). The use of this non-invasive analysis allows for stratification of patients by their probability of achieving a favorable clinical response, laying the groundwork for designing strategies aimed at restoring microbial homeostasis and enhancing chemosensitivity (3).

Nevertheless, these intensive cytotoxic regimens damage the epithelial barrier and disrupt oral eubiosis, favoring the development of acute complications such as ulcerative mucositis. This condition increases the risk of systemic infections and is often associated with dose reduction or delays in treatment cycles (4). In the face of this challenge, Petropoulou et al. demonstrated through a randomized clinical trial the effectiveness of telemedicine and mobile applications (e-Health) in the dental field (5). Patients under this interactive remote monitoring displayed a significant reduction in the frequency and severity of oral mucositis, as well as better control of gingivitis and periodontal disease compared to conventional care (5). This care model aligns with what was reported by Mooney et al., (6) who showed that electronic symptom monitoring systems based on patient-reported outcomes (ePROs) reduce symptom burden and improve adherence to chemotherapy (6). Thus, the study by Petropoulou et al. complements Fu et al.‘s approach, showing that preserving oral health in oncology patients requires a dual strategy: microbiological stratification before initiating treatment and real-time digital outpatient support to ensure the continuity of systemic therapy (3, 5).

On another front, optimization of supportive care extends to pediatric oncology, where cytotoxic regimens achieve high survival rates at the cost of chronic sequelae. Among these adverse effects, iron overload secondary to repeated transfusional support constitutes a critical risk factor that compromises tissue homeostasis. In this area, Kamış et al. assessed accumulation of this metal in a pediatric cohort by comparing two indicators: serum ferritin levels and findings from magnetic resonance imaging (MRI) using the T2 star technique (7). Their study demonstrated a notable clinical discrepancy; while blood ferritin behaved as an accessible but nonspecific parameter, being altered by concurrent inflammatory processes, the MRI technique showed high diagnostic accuracy in detecting real iron deposition in hepatic parenchyma (7). Consequently, conventional monitoring with plasma biomarkers is insufficient for predicting the real risk of overload. Kamış et al.‘s work thus underscores the need to incorporate advanced imaging techniques to initiate iron chelation in a timely manner and prevent chronic organ damage in these children (7).

Finally, the sequelae of pediatric oncological therapies are manifested not only systemically, but also permanently compromise maxillofacial growth. In this respect, Krommenhoek et al. presented the radiological tool DENTALE to assess and score late dental and skeletal alterations in survivors of head and neck rhabdomyosarcoma (8). This system precisely records anomalies such as agenesis, microdontia, and deficiencies in root development caused by radiotherapy and chemotherapy during childhood (8). This type of tool directly connects oncological treatment with specialized dental practice, facilitating comprehensive therapeutic planning (8).

In conclusion, the articles in this Research Topic demonstrate that current oncological success must include the preservation of long-term quality of life. This requires a continuous model linking initial microbiological stratification, interactive digital monitoring during active treatment, and radiological monitoring of chronic sequelae, ensuring truly personalized clinical support.
